# Validation of a Vision-Guided Mobility Assessment for *RPE65*-Associated Retinal Dystrophy

**DOI:** 10.1167/tvst.9.10.5

**Published:** 2020-09-03

**Authors:** Neruban Kumaran, Robin R. Ali, Nick A. Tyler, James W. B. Bainbridge, Michel Michaelides, Gary S. Rubin

**Affiliations:** 1 UCL Institute of Ophthalmology, University College London, London, UK; 2 Moorfields Eye Hospital, London, UK; 3 Guy's and St. Thomas’ NHS Foundation Trust, London, UK; 4 NIHR Moorfields Biomedical Research Centre, Moorfields Eye Hospital, London, UK; 5 Department of Civil, Environmental and Geomatic Engineering, University College London, London, UK

**Keywords:** LCA, retinal dystrophy, RPE65, night vision, mobility

## Abstract

**Purpose:**

To validate a vision-guided mobility assessment for individuals affected by *RPE65*-associated retinal dystrophy (*RPE65*-RD).

**Methods:**

In this comparative cross-sectional study, 29 subjects, comprising 19 subjects with *RPE65*-RD and 10 normally-sighted subjects undertook three assessments of mobility: following a straight line, navigating a simple maze, and stepping over a sidewalk “kerb.” Performance was quantified as the time taken to complete each assessment, number of errors made, walking speed, and percent preferred walking speed, for each assessment. Subjects also undertook assessments of visual acuity, contrast sensitivity, full-field static perimetry, and age-appropriate quality of life questionnaires. To identify the most relevant metric to quantify vision-guided mobility, we investigated repeatability, as well as convergent, discriminant, and criterion validity. We also measured the effect of illumination on mobility.

**Results:**

Walking speed through the maze assessment best discriminated between *RPE65*-RD and normally-sighted subjects, with both convergent and discriminant validity. Walking speed also approached statistical significance when assessed for criterion validity (*P* = 0.052). Subjects with *RPE65*-RD had quantifiably poorer mobility at lower illumination levels. A relatively small mean difference (−0.09 m/s) was identified in comparison to a relatively large repeatability coefficient (1.10 m/s).

**Conclusions:**

We describe a novel, quantifiable, repeatable, and valid assessment of mobility designed specifically for subjects with *RPE65*-RD. The assessment is sensitive to the visual impairment of individuals with *RPE65*-RD in low illumination, identifies the known phenotypic heterogeneity and will furthermore provide an important outcome measure for *RPE65*-RD.

**Translational Relevance:**

This assessment of vision-guided mobility, validated in a dedicated cohort of subjects with *RPE65*-RD, is a relevant and quantifiable outcome measure for *RPE65*-RD.

## Introduction

Leber congenital amaurosis (LCA) is an inherited retinal disease which presents as a progressive rod-cone dystrophy.^1^ It affects up to one in 33,000 live births, and 25 genes have been identified to date as causing approximately 70% to 80% of cases. Recessive *RPE65*-associated retinal dystrophy (*RPE65*-RD) is thought to account for approximately 5% to 10% of LCA and 1% to 2% of retinitis pigmentosa, and successful gene replacement has been demonstrated in phase I/II and III clinical trials.^2^^–^^9^ This has led to *RPE65*-RD becoming the first ocular condition for which a United States Food and Drug Administration (FDA)– and European Medicines Agency (EMA)–approved treatment (Luxturna, Spark Therapeutics Inc, Philadelphia, PA, USA) is now available. Of note, the primary outcome of the phase III trial demonstrating efficacy of this gene therapy was improved mobility in dim light after intervention.^7^

A total of three groups have used mobility performance tasks to demonstrate improved mobility at low illumination levels following gene therapy intervention for *RPE65*-RD.^10^^–^^12^ Jacobson et al.^12^ describe the use of an indoor obstacle course in a room (4.6 m × 27 m) with wall segments tethered to the ceiling and floor-level obstacles. In this study, mobility was quantified as the number of errors made during the course at specified light levels. Maguire et al.^11^ initially describe a mobility assessment, which has since been further developed by Chung et al.^13^ into a multiluminance mobility test (MLMT) for patients with inherited retinal dystrophies. The MLMT consists of a much smaller (2 m × 3.6 m) white cloth that is divided into 30-cm squares, where each square has either a black arrow, to indicate the direction of travel, or an obstacle to be avoided, with subsequent performance being graded as either a pass or a fail. Interestingly, while Chung et al.^13^ suggest their findings demonstrate adequate validity, their assessments appear limited by strong ceiling effects, as exemplified by all unaffected individuals and some affected individuals passing the assessment. We have previously used a very similar assessment to the one described herein, to also demonstrate improved mobility in dim light following intervention.^10^ In addition, a fourth group has used a mobility maze in their *RPE65*-RD gene therapy trial, with subjects assessed at two light levels.^6^ However, no change was identified after intervention, and no description is provided of their assessment.

Visual function tests can predict visual performance under real world conditions,^14^^,^^15^ and there is increasing interest in developing standardized assessments of visual function. Vision-guided mobility is one such area of interest and can be defined as the ability to use sight to move through the environment in a safe and efficient manner.

Several studies have investigated mobility performance in people with sight-impairment. In a landmark study, Marron and Bailey^16^ used both an outdoor course (that included an entire city block) and an indoor course (long corridor measuring 12.2 m × 2.4 m), both of which included obstacles, to evaluate mobility in 19 visually-impaired subjects. Performance was quantified as the reciprocal of the number of errors made by the subjects. Performance was observed to correlate poorly with visual acuity (VA), but more strongly with contrast sensitivity and visual field (VF) preservation, particularly peripheral VF. Several studies ranging from small laboratory investigations to large population-based studies have confirmed that mobility performance is associated with VF, independent of VA.^17^^–^^19^

In preparation for the evaluation of a retinal implant for people with very low vision, Velikay-Parel et al.^20^ created a mobility course situated in an 11.2 m × 2.8 m corridor and instructed the subjects to traverse a serpentine path around mobile screens while avoiding various obstacles. Interestingly, transit time was associated with VF size, and to a lesser extent with VA, whereas errors were not significantly associated with VA or VF.

Additionally, Geruschat et al.^21^ describe the use of a 18.3 m × 1.4 m hallway consistently illuminated, seeded with a variety of obstacles as one aspect of an orientation and mobility assessment of an optobionic retinal implant. Ultimately, no significant difference was noted in mobility before and after implantation when assessed with the number of errors made or time to complete the assessment. Interestingly, the group highlight the importance in identifying a sensitive metric to measure significant change in mobility.

Furthermore, Nau and colleagues^22^ describe a mobility assessment for people with very low vision who were being evaluated for the BrainPort sensory substitution device, which was undertaken in a 12 m × 2 m corridor containing obstacles. Performance was quantified using the percent preferred walking speed (PPWS) metric, defined as the time taken to walk through a course with obstacles, divided by the time to traverse the corridor without obstacles. Here we describe the validation of our current mobility assessment used in both the phase I/IIa *RPE65*-RD gene therapy trial (clinicaltrials.gov identifier: NCT02781480) and prospective *RPE65*-RD natural history study (NCT02714816).

## Methods

### Subjects

Ten unaffected individuals (five adults and five children) and 19 molecularly confirmed recessive *RPE65-*RD–affected individuals (10 adults and nine children) undertook the mobility assessment and assessments of visual function. Assessment of mobility, best corrected visual acuity (BCVA), contrast sensitivity, full-field static perimetry were all undertaken monocularly, to give results greater context in preparation for future trials, where intervention is likely to be one eye at a time. The study protocols adhered to the Tenets of the Declaration of Helsinki and received local approvals. Informed consent was obtained from all adults, whereas informed consent and assent were obtained from parents and children, respectively, before enrollment.

### Mobility Course

**Figure 1.**
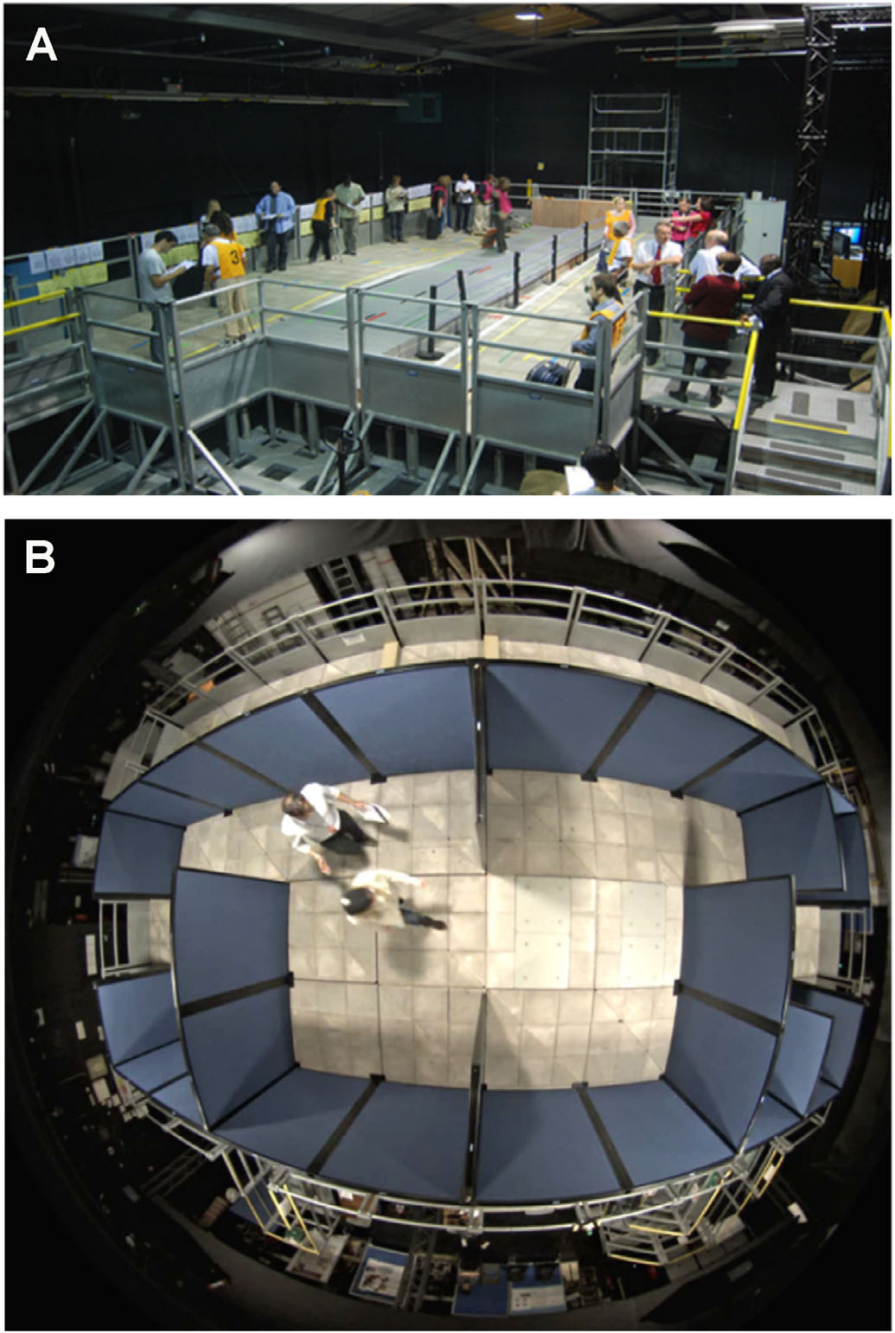
PAMELA Facility at University College London, London, UK. (A) raised platform. (B) “Fisheye” view from overhead camera showing the “maze” assessment being completed by a participant and followed by a grader.

**Figure 2.**
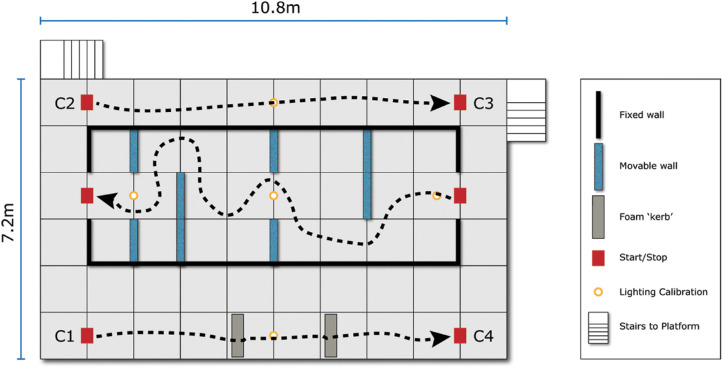
Schematic showing configuration of straight-line walk, maze, and kerb assessments at the PAMELA facility with potential courses shown with *dashed lines*.

All assessments were performed at the Pedestrian Accessibility Movement Environment Laboratory (PAMELA), at University College London (UCL), London, UK (Fig. 1). The mobility assessment was initially developed for use in the UCL-sponsored *RPE65*-RD gene therapy trial.^2^ This was subsequently replicated and improved to the assessment described herein. PAMELA provides a simulated sidewalk environment, where light levels can be controlled over a range from 1 to 15,000 lux. PAMELA is configured as a raised 10.8 m × 7.2 m platform, arranged into three sections as follows (as shown in Fig. 2):

1. A 10 m straight-line walk (“straight”) represented between points C2 and C3 in Figure 2, with the corresponding start/stop locations indicated.
2. A 13 m maze in the middle section (“maze”) with corresponding stop/start locations. The moveable barriers can be repositioned to create multiple maze configurations.
3. Another 10 m straight-line walk with two foam blocks to simulate sidewalk kerbs (“kerb”), with corresponding start/stop locations (C1 and C4, Fig. 2). The foam blocks were a light-yellow color with chromaticity co-ordinates 0.46, 0.42, and a Weber contrast of 0.32 between the “kerb” and the pavement. These foam blocks measured 120 cm (width) × 20 cm (depth) × 13cm (height).

The partitions used were modular wall panels measuring 1.8 m × 1.2 m, covered with navy blue fabric, and the frames, supports and feet painted black. Computer-controlled LED panels were used to achieve illumination levels of 256, 64, 16, 4, and 1 lux, at the floor level of the platform. These were selected to approximate a variety of different, common real-world illumination levels (Table 1).

**Table 1.** Illumination Levels and Equivalent Real-World Environments^39^^,^^40^

Illumination Level (Lux)	Equivalent Real-World Environment
1	Deep twilight
4	Residential street lighting
16	Twilight
64	Car park
256	Office work

### Procedure

Each eye of each subject was tested monocularly, with the contralateral eye occluded. Subjects were not allowed to use a cane to aid mobility. The panels were configured in one of four configurations, and each configuration was tested in a forward and reverse direction, to provide a total of eight different layouts. For each change of the maze configuration the kerb location was changed by a distance of up to 1 m. The light levels were calibrated daily (to within 20% of the nominal value at the center of the platform), with readings also taken at each of the marked positions in Figure 2. The “straight” assessment was undertaken first, followed by the “maze” and subsequently by the “kerb” assessment.

Tests were conducted in decreasing levels of illumination. The decision was made to assess subjects at decreasing light levels (starting with the brightest light level) for three reasons. Firstly, to lessen the anxiety of affected subjects, with the knowledge that poor vision in dim light is the hallmark of *RPE65*-RD. Secondly, decreasing the light levels allowed us to decide whether to proceed with more difficult trials. Finally, decreasing light levels make more efficient use of adaptation time, thereby making the experiment shorter for the participants.

At each light level, the same configuration of the maze was used for every subject. Subjects adapted to the ambient illumination level for 10 minutes before each test. During that period, the panels were moved into place with the illumination level set to the predetermined value. Subjects were provided verbally with standardized instructions for each section of the course (Supplementary material). The subject was then guided into position and when ready, instructed to walk to the end of the section at a normal comfortable pace while avoiding contact with the barriers. The experimenter followed along behind to ensure the subject's safety and documented both the time taken to complete the course (course time) using a stop watch, and the number of errors made. An error was recorded if the participant made contact with a barrier, stumbled, or lost their way and had to be reoriented by the experimenter. An overhead camera was used to record the participant's path through the course, allowing potentially ambiguous results to be reviewed.

The course length (meters) was divided by time to complete the course (seconds) to give walking speed (meters/second; m/s). Walking speed for the maze portion was divided by walking speed for the straight unimpeded section, and multiplied by 100, to give a percent-preferred-walking-speed (PPWS).

### Other Assessments of Visual Function

Best corrected LogMAR visual acuity (BCVA), contrast sensitivity, full-field static perimetry, and quality of life (QoL) were also assessed in affected *RPE65*-RD subjects. All unaffected subjects reported normal vision. BCVA was assessed monocularly with an Early Treatment Diabetic Retinopathy Study chart, illuminated with a Precision Vision lightbox to 150cd/m^2^ (Precision Vision, Woodstock IL, USA). Contrast sensitivity (CS) was assessed monocularly using the Pelli-Robson chart at 1m, with room lighting allowing a chart luminance of 100 cd/m^2^. The LogCS value was subsequently calculated. Full-field static perimetry was performed using the Octopus 900 (Haag Streit AG, Köniz, Switzerland), GATE testing strategy (Haag Streit AG), stimulus size V, a 200 ms presentation, and a radially-designed, centrally condensed grid of 164 test locations that extended radially to 80° temporally, 67° inferiorly, and 55.5° nasally and superiorly. The vendor software (EyeSuite) subsequently produced a mean sensitivity value in decibels. Furthermore, Visual Field Modeling and Analysis software (VFMA, Office of Technology Transfer and Business Development [OHSU], Portland, OR, USA) was used to calculate the volume of the total hill of vision (V_TOT_) quantified in decibel-steradian (dB-sr), using previously reported methods.^23^^–^^25^

QoL was assessed using the Impact of Vision Impairment (IVI) Questionnaire, with adults completing the adult version (IVI)^26^ and children the child version (IVI-C.)^27^ In particular, we investigated the following two questions as they were the questions most pertinent to mobility. Adults were asked “In the past month, how often has your eyesight made you go carefully to avoid falling or tripping?” and given a choice of the following responses; 1—a lot, 2—a fair amount, 3—a little, 4—not at all, 8—don't do this for other reasons. The responses were subsequently dichotomized into substantial problems (response 1) and some/no problems (responses 2, 3, and 4), for analytical purposes. Children were asked, “Are you confident that you can move around safely in places you don't know at night time?” and given a choice of the following responses; 5—Always, 4—Almost always, 3—Sometimes, 2—Almost Never, 1—Never, or No for other reasons). These responses were subsequently dichotomized into substantial problems (response 1) and some/no problems (responses 2, 3, 4 and 5), for analytical purposes.

### Statistical Analysis

To identify the best metric for characterizing mobility performance, a receiver operating characteristic (ROC) curve analysis was performed using the ROCCOMP procedure in Stata (Statacorp, College Station, TX, USA). This ROC curve assessed the right eyes of all subjects by using data across all light levels and comparing affected to unaffected subjects.

To investigate repeatability, the repeatability coefficient, mean difference, and upper and lower limits of agreement were calculated. Statistical analysis was undertaken only in right eyes of *RPE65*-RD subjects and was performed using Stata (StataCorp). The distribution of data was evaluated using the Shapiro-Wilk test, confirming that there was no significant departure from normal distribution. The “forward” and “reverse” runs of each assessment were designated as runs 1 and 2 to assess the above. The repeatability coefficient was calculated as: 1.96 × √2 x the within-subject standard deviation.^28^ The within-subject standard deviation was calculated using an analysis of covariance (ANCOVA) model on walking speed for the maze portion against two repeated runs (first run compared to second run in the same eye), where run was the random effect. Furthermore, the mean difference and upper and lower limits of agreement were derived from a Bland-Altman plot.^29^

Convergent, discriminant and criterion validity were investigated using a mixed-effects linear regression analysis with terms adjusting for light level on data from right eyes of *RPE65*-RD subjects. The effect of illumination is described in terms of the mean and standard error, both calculated from data from right eyes only. To minimize the clustering effect of using data from both eyes, it was decided at the end of the study to only analyze results from the right eye of all subjects, as described above.

## Results

### Patient Demographics

Ten normally-sighted subjects and 19 molecularly confirmed *RPE65-*RD affected subjects undertook the mobility assessment. The demographics for both cohorts is presented in Table 2.

**Table 2.** Demographics Showing Number of Affected and Unaffected Individuals, Age (Median and Interquartile Range; IQR), Visual Acuity (VA; Median and Interquartile Range; IQR) and Gender, for Both Adults and Children

	Adults					Children				
	Number	Number of Eyes	Age Median (IQR)	VA Median (IQR)	Gender	Number	Number of Eyes	Age Median (IQR)	VA Median (IQR)	Gender
Affected	10	20	20 (19–21)	0.7 (0.6–1.15)	6 male and 4 female	9	18	11 (9–11)	0.7 (0.7–0.9)	3 males and 6 females
Unaffected	5	8	38 (30–38)	x	3 male and 2 female	5	10	8 (7–8)	x	1 male and 4 female

Visual acuity was not assessed for unaffected individuals because they reported themselves to be normally sighted. Two unaffected adult subjects undertook the assessment with one eye alone, despite not having any ocular pathology.

### Identifying the Most Appropriate Metric for Mobility

**Figure 3.**
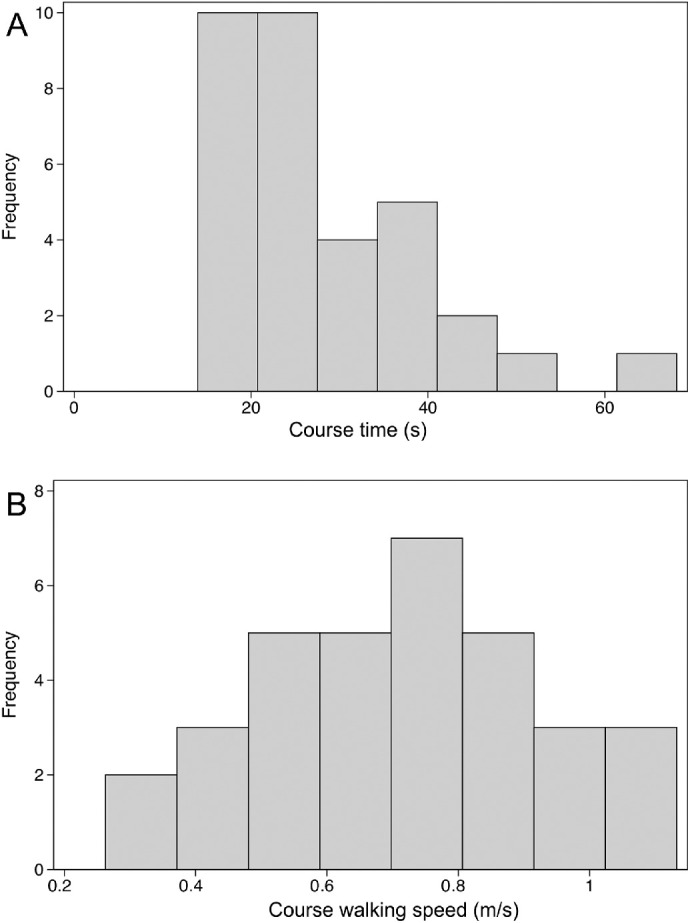
Histograms showing distribution of course time (A) and walking speed (B) in right eyes of affected subjects. Course time shows a skewed distribution which is largely corrected for by converting course time into walking speed.

To identify the most appropriate metric for a valid and repeatable test, we evaluated course time, walking speed, error count, and percent preferred walking speed, as defined above. Course time gave a highly skewed distribution, which was largely corrected by converting to walking speed (calculated by dividing the length of the course by the course time), as demonstrated in Figure 3.

**Figure 4.**
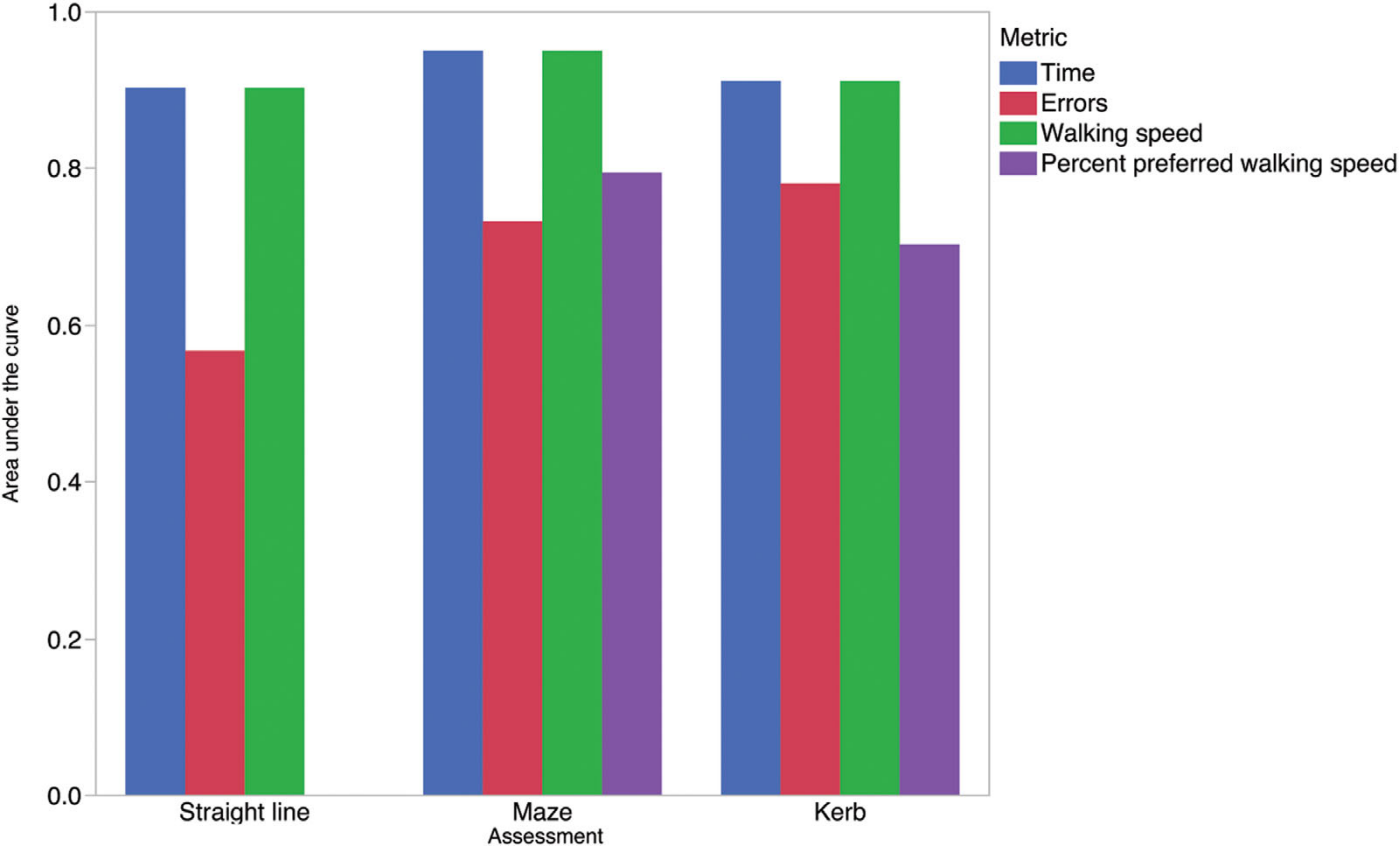
Bar chart demonstrating area under the ROC curve for each metric for each assessment. Area under the ROC curve was assessed in right eyes of all subjects by using data across all light levels and comparing affected to unaffected subjects. The closer the AUC to 1 the greater the ability of the metric to discriminate between *RPE65*-RD subjects and unaffected individuals. This demonstrates that walking speed and time to complete the maze assessment best discriminate *RPE65*-RD subjects from normally-sighted subjects.

To identify the best metric for characterizing mobility performance, we conducted an ROC curve analysis that describes how well a measure makes a binary discrimination between the unaffected and *RPE65*-RD subjects. Discrimination ability is quantified by the area under a ROC curve (AUC) and ranges from 0.5 (discrimination no better than chance) to 1.0 (perfect discrimination). Figure 4 and Supplementary Table S1 demonstrate the AUCs for each mobility metric and identify walking speed and course time through the maze assessment as the most accurate metrics for discriminating between these two cohorts. Given the less skewed distribution of walking speed and equal AUCs of both walking speed and time (through our maze assessment), we limit our subsequent analyses to the walking speed outcome measure.

### Repeatability

**Figure 5.**
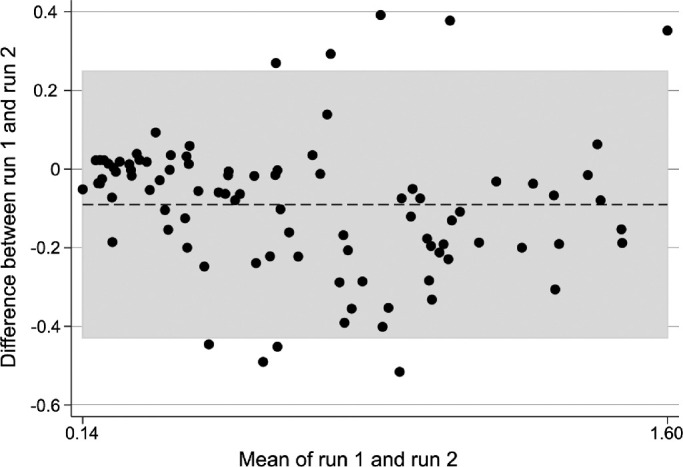
Bland Altman plot showing agreement of walking speed (m/s) between two runs of the maze assessment, in right eyes of affected subjects at all light levels. *Dashed line* represents average mean difference and *shaded area* represents limits of agreement.

As all outcome assessments are subject to measurement error, it is important to understand the sources of measurement error, so that they may be accounted for in the interpretation of the data. “Repeatability” refers to measurement error for two (or more) identical measurements, such as two measurements of VA taken by the same examiner and using the same charts and procedures. Reliability refers to the measurement error for two measurements of the same outcome that differ in some way, such as different examiners, different instruments or different procedures. We investigated the repeatability (of two “runs” within one visit) of our maze assessment using the repeatability coefficient, the mean difference, and the 95% limits of agreement (the latter two derived from Bland-Altman plots), as demonstrated in Table 3 and Figure 5. There was no substantial difference in average walking speed between test and retest, indicating that there was little or no learning effect for the task.

**Table 3.** Repeatability Analysis

Number of Subjects	Repeatability Coefficient (m/s)	Mean Difference (m/s)	Lower Limit of Agreement (m/s)	Upper Limit of Agreement (m/s)
19	1.10	−0.09	−0.43	0.25

Shown are the number of subjects, repeatability coefficient, mean difference, and upper and lower limits of agreement.

One must, however, exercise caution when interpreting the mean difference and limits of agreement because neither remains constant over the range of walking speeds elicited by our task. Notably, we found relatively large repeatability coefficient (1.10 m/s) and limits of agreement (−0.43 m/s to 0.25m/s) as demonstrated in Table 3 in comparison to the range of mean walking speeds (0.14 m/s to 1.6 m/s) as demonstrated in Figure 5.

### Validity

Many different forms of validity exist, of which we address three: convergent, discriminant and criterion validity. Convergent and discriminant validity are two opposing aspects of construct validity. Convergent validity tests that constructs expected to be related, are truly related. Conversely, discriminant validity tests that constructs expected to be unrelated, are indeed unrelated. Given findings of other groups, described above, we expected to show a correlation between mobility and both the mean retinal sensitivity (MS) and the volume of the total hill of vision (V_TOT_) – measures of VF, and less of a correlation, or no correlation, between mobility and both contrast sensitivity (CS) and VA. With this in mind, we examined the effect of VA, CS, MS and V_TOT_ on walking speed, adjusting for light level, using mixed effects linear regression analysis. This identified that MS (*P* = 0.022) and V_TOT_ (*P* = 0.022) could statistically significantly predict walking speed. More specifically, walking speed for maze portion positively correlates with MS and V_TOT_ under the lower light levels specifically 16 and 4 lux (Supplementary Figs. S2, S3), suggesting faster walking speed with increased retinal sensitivity. In comparison, no such correlation was identified between walking speed and VA (*P* = 0.340) or CS (*P* = 0.433).

The third type of validity we investigated, criterion validity, assesses whether a measurement is related to an indicator. We assessed criterion validity by comparing walking speed to subjects’ dichotomized responses on a quality of life questionnaire. We used binomial logistic regression to investigate the relationships of walking speed and light level with the participants' reported difficulties with mobility. Walking speed approached significance (*P* = 0.052) and was positively associated with affected subjects’ perceived difficulties with mobility, thereby supporting criterion validity of our mobility assessment.

### Effect of Different Illumination Levels

**Figure 6.**
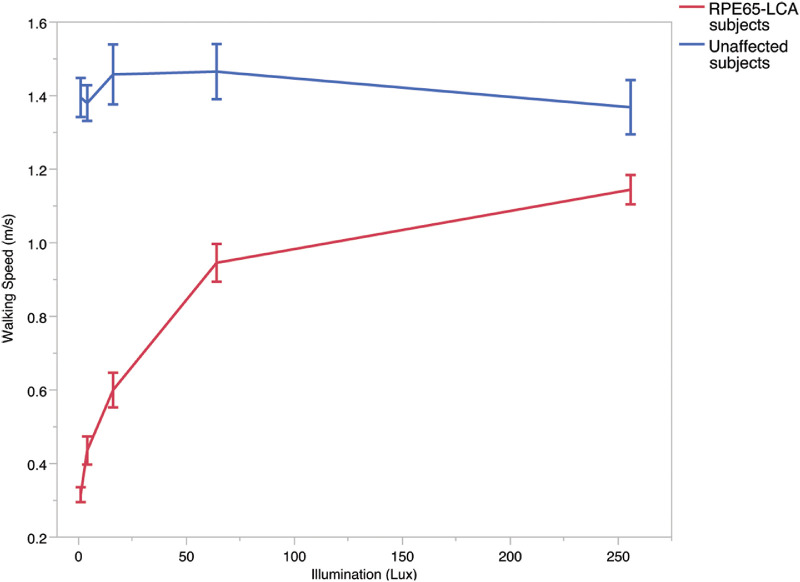
Effect of illumination on walking speed in *RPE65*-RD subjects and normally-sighted subjects. Shown is a *line graph* of the mean walking speed, through the maze assessment, at each illumination with errors indicating standard error. This demonstrates a relatively consistent walking speed across light levels in normally-sighted subjects, in comparison to *RPE65*-RD subjects who walk at a much slower speed at lower light levels with an improvement in walking speed at higher light levels.

Finally, we examined the test's responsiveness to illumination level by comparing the performance of affected and unaffected individuals under a range of light levels. Normally-sighted subjects performed consistently well at all light levels (Fig. 6). Subjects with *RPE65*-RD, in contrast, walked more slowly at all light levels; their walking speed was faster at higher light levels but did not match that of normally-sighted subjects even at 256 Lux.

## Discussion

In this study, we showed that it is feasible to construct a highly standardized test of vision-guided mobility and that through using a quantifiable metric this assessment (specifically the maze section) is repeatable, valid and responsive to illumination levels, and moreover specific to subjects with *RPE65*-RD.

It has long been suggested that navigation correlates strongly with VF and poorly with VA.^16^ In keeping with this, our assessment demonstrates both convergent and discriminant validity respectively, showing a correlation with visual field (measured with mean retinal sensitivity, *P* = 0.022 and volume of the total hill of vision, *P* = 0.0222), but no correlation with VA (*P* = 0.340). Interestingly, Geruschat et al.^19^ investigated mobility in a cohort of 25 patients with retinitis pigmentosa (RP), which is similar to LCA, with progressive rod and subsequent cone photoreceptor degeneration, but with later onset and less rapid progression. They too identified that subjects with RP had a slower walking speed than normally-sighted subjects, and furthermore, contrast sensitivity and VF accounted for nearly 70% of the variance of walking speed. Additionally, they also identified that RP subjects reported greater dissatisfaction with mobility, which was closely associated with walking speed. Data using our assessment are in agreement with these findings, with walking speed performance also associated with reported difficulties with mobility (*P* = 0.052). Furthermore, this group also identified worse mobility at lower illumination levels (defined by a greater number of errors) in subjects with RP.

In assessing the repeatability of our assessment, we found relatively large coefficient of repeatability, lower and upper limits of agreement. We attribute this to the phenotypic heterogeneity seen in *RPE65*-RD as established in our group's previous studies of both retinal structure and function in *RPE65*-RD.^24^^,^^30^^,^^31^ Furthermore, we suggest that this demonstrates the importance of multiple baseline assessments per subject should this assessment be used in an interventional setting. Such multiple assessments at baseline have proven to be valuable in natural history studies and interventional studies using retinal sensitivity as an endpoint in both *RPE65*-RD and other inherited retinal diseases.^5^^,^^24^^,^^32^ It could be extrapolated further to suggest the importance of identifying repeatability per subject, per eye, in an interventional setting, if this value is used to define a clinically meaningful change.

In 2018, the FDA and subsequently EMA, licensed the first ocular gene therapy (Luxturna) after a phase III trial demonstrating improved navigation in dim light using a mobility assessment (MLMT) described by Chung et al.^13^ There are significant differences between the MLMT and our assessment. First, while Chung et al.^13^ validate their tool with subjects diagnosed with a broad range of IRDs, we used a dedicated cohort of molecularly confirmed *RPE65*-RD subjects. We made this decision given the vast phenotypic heterogeneity of IRD. Second, Chung and colleagues^13^ describe a much smaller scale assessment using a 2.1 m × 3.6 m floor cloth consisting of 0.3 m squares with arrows. In contrast, by developing an assessment more in keeping with other specialist groups in the field of orientation and mobility, such as Velikay-Parel et al.^20^ (11.2 m × 2.8 m) and Marron and Bailey^16^ (12.4 m × 2.4 m), we established our assessment using a 10.8 m × 7.2 m platform, consisting of sidewalk slabs.^13^ Third, Chung et al.^13^ describe the use of a binary pass-fail outcome based on a combination of speed and accuracy, weighted for illumination. We decided against using such a binary measure both because of the ceiling effect it can induce, and moreover, because much data exists confirming that speed, time taken, patient preferred walking speed or error measurements are essential, well-recognized methods of scoring in navigation assessments.^16^^,^^33^^,^^34^

Interestingly a further mobility assessment, the Ora-VNC (Ora Therapeutics, Andover, MA, USA) has also been developed for interventional clinical trials.^35^ This assessment aims to assess mobility at varied light levels using marked paths with a series of physical obstacles. To our knowledge little has been published on its validation; however, it is understood to have been conducted comparing unaffected individuals to subjects with goggle-simulated visual impairment for modeling of mild or severe RP.^36^ Similar to the MLMT the use of marked paths raises questions as to its applicability in “real-world” mobility.

Furthermore, we looked to investigate whether a straight line assessment with the addition of 2 “kerbs” could be used to quantify mobility. This was of particular interest because difficulty with floor level elevation is commonly reported by patients,^37^ with, however, nothing in the literature, to our knowledge, reported on its use as an assessment of mobility. Notably, we found this to be a good assessment of mobility (using the walking speed metric) in differentiating affected and unaffected individuals (AUC = 0.91); but, however, not as good as the maze assessment, the assessment we ultimately chose to use.

Nevertheless, our study has limitations. One key limitation, as with all assessments of navigation, is the difficulty in recreating a realistic environment. Ideally, such assessments would be best performed outdoors, because such courses may better reflect real-life vision-guided navigation. However, outdoor environments often prove difficult (if not impossible) to control reliably. Similarly, given our aim was to validate this assessment for *RPE65*-RD specifically, further studies may be required to evaluate the utility in other IRDs. We do however expect that this setup could readily be used for conditions such as RP where patients also struggle in dim light.

Another challenge and limitation of the study was assessing criterion validity using self-reported measures. Ultimately, we used two questions from the IVI and IVI-C questionnaires. A key difference between the two existed, in that the adult version of the question did not specify a time of day, whereas the child version did. The question also did not investigate the daily travel habits of our subjects or if they regularly used a cane for daily travel. This challenge ultimately stems from the fact that visual loss and young age of onset of *RPE65*-RD is markedly different from the ocular disorders (age-related macular degeneration, cataract, glaucoma) used to validate both health-related and vision-related quality of life questionnaires. Furthermore, the young age of onset with relatively fast rate of progressive visual loss noted in *RPE65*-RD makes it difficult to use QoL questionnaires developed to assess mobility in more common inherited retinal diseases such as RP.^38^

Additionally, in our study, the assessment was undertaken monocularly. Although this may help validate the assessment in preparation for its use in interventional trials, it is also a limitation because subjects would use binocular vision in real-life mobility tasks. Finally, unaffected subjects did not undertake assessments of visual function other than the mobility assessment. This decision was made because all unaffected subjects reported no visual concern; however, measurement of unaffected visual function would have further supported the findings herein.

In summary, we have developed and refined a vision-guided navigation assessment that is repeatable and relevant to reported navigation ability in the real world. This assessment (specifically the maze portion of the course) can be used to quantify changes in mobility using a continuously scaled metric that correlates with retinal sensitivity for a specific group of patients. Such a robust assessment of vision guided mobility will be valuable in both understanding the natural history of *RPE65*-RD and measuring the impact of intervention.

## Supplementary Material

Supplement 1
