# Supplementary material for: Validation of a Vision-Guided Mobility Assessment for RPE65-Associated Retinal Dystrophy
Source: Transl Vis Sci Technol. 2020 Sep 3;9(10):5. doi: 10.1167/tvst.9.10.5 (PMC7476654; doi:10.1167/tvst.9.10.5)
Supplement: Supplement 1 [file tvst-9-10-5_s001.pdf]

Validation of a vision-guided mobility assessment for *RPE65*-associated retinal dystrophy – Supplementary Material

Neruban Kumaran, Robin R Ali, Nick A Tyler, James W B Bainbridge, Michel Michaelides, Gary S. Rubin.

The following standardized instructions were read out to the subjects.

General instructions

For adults: "The experiment consists of three sections. For each section we will give you specific instructions and ask you to walk the length of the platform at a normal comfortable pace. We will be monitoring your progress with various cameras. Please try to avoid touching any of the obstacles that you may come across. I will be following close behind you to make sure you are safe. The experiment will be repeated up to 5 times, under various lighting conditions and with each eye covered."

For children: "This exercise is divided into 3 sections and for each section we will tell you what to do. We will ask you to walk along a platform in the way you usually walk and we will record it. Don't run, just walk normally and try not to touch any of the walls. I will be just behind you to make sure you are safe. You will be asked to repeat this exercise up to 5 times, in different lights with each eye covered."

Section 1 ('Straight line walk')

For adults: "When I tell you to begin, please walk straight down the length of the pavement to the corner and stop. Please walk at a normal comfortable pace. Do you understand these instructions?" [Answer any questions]

For children: "When I say 'go' you can walk ahead to the far corner and then stop. Is that OK? Do you want to ask me anything?" [Answer any questions]

Section 2 ('Maze')

For adults: "In front of you is a simple maze. There are four rows of barriers painted dark blue. Sometimes there will be a gap in the middle of the barrier that you must walk through and sometimes you will have to walk around to the left or right of the barrier to get past it. When I tell you to begin, please walk to the other end of the pavement taking the shortest route you can. Please keep walking until I tell you to stop. Remember to try not to touch the barriers and walk at a normal comfortable pace. Do you understand these instructions?" [Answer any questions]

For children: “In front of you is a simple maze with four rows of blue barriers. When you see a gap in the middle of the barrier please walk through it. Sometimes you may need to go around it to get past it. When I tell you ‘go’ you can walk to the end of the pavement taking the shortest way you can and walk until I tell you to stop. Try not to touch anything. Is that OK? Do you want to ask me anything?” [Answer any questions]

<u>Section 3 (‘Kerb’)</u>

For adults: “When I tell you to begin, please walk straight down the length of the pavement to the opposite corner and stop. As you walk along please notice that there are two foam blocks in the path. As soon as you come to them please step over and continue to the end of the pavement. Do you understand these instructions?” [Answer any questions]

For children: “When I say ‘go’ you can walk ahead to the corner on the other side and then stop. There are two blocks made of foam in your way, could you step over them, trying not to touching them and carry on? Is that OK? Do you want to ask me anything?” [Answer any questions]

Supplementary Tables

**Assessment**	**Metric**	**Area Under Curve**
Straight line	Time	0.90
Maze	Time	0.95
Kerb	Time	0.91
Straight line	Errors	0.57
Maze	Errors	0.73
Kerb	Errors	0.78
Straight line	Walking speed	0.90
Maze	Walking speed	0.95
Kerb	Walking speed	0.91
Maze	PPWS	0.80
Kerb	PPWS	0.70

Supplementary Table 1: Area under the curve (AUC) for each metric of assessment. The closer the AUC to 1 the greater the ability of the metric to discriminate between *RPE65*-LCA subjects and unaffected individuals.

Supplementary Figure Legends

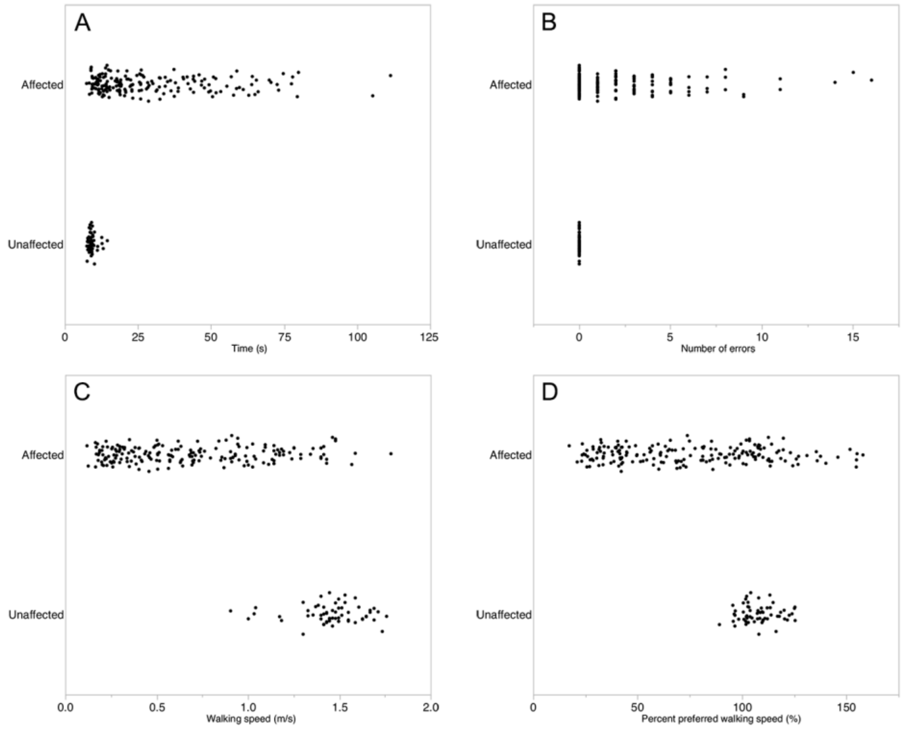

Supplementary Figure 1: Scatter plots for maze assessment. Shown are scatter plots for the metrics of time (A), errors (B), walking speed (C) and percent preferred walking speed (D) for the right eyes of affected individuals undertaking the 'maze' assessment.

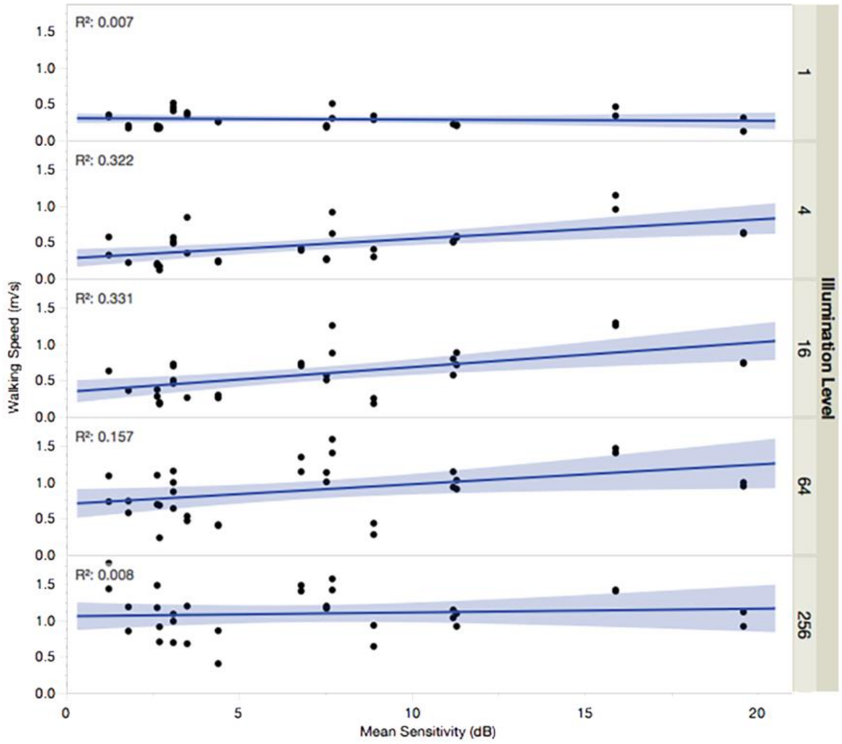

Supplementary Figure 2: Walking speed compared to mean sensitivity by each illumination level. Show are scatterplots and linear regression lines of walking speed (m/s) against mean sensitivity (dB) by each illumination level (lux) with associated R^2 values, for the right eyes of *RPE65*-LCA subjects.

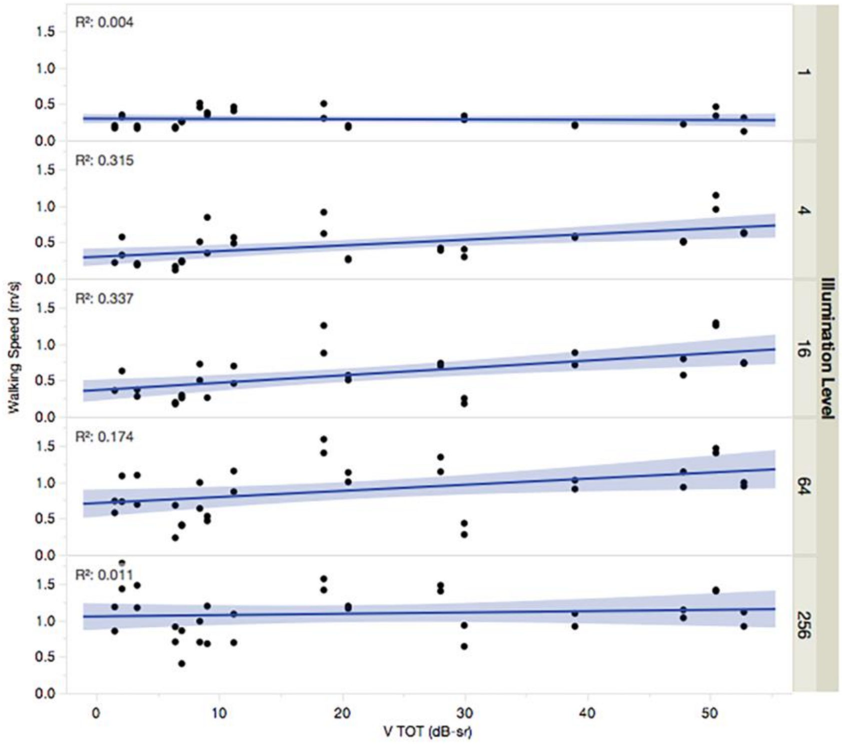

Supplementary Figure 3: Walking speed compared to total hill of vision by each illumination level. Show are scatterplots and linear regression lines of walking speed (m/s) against total hill of vision (V TOT; dB-sr) by each illumination level (lux) with associated R^2 values, for the right eyes of *RPE65*-LCA subjects.
